# Direct detection of Gram-negative bacilli and extended-spectrum β-lactamase producers in positive blood culture specimens using MALDI-TOF MS

**DOI:** 10.3389/fcimb.2025.1701423

**Published:** 2025-11-21

**Authors:** Kosuke Kosai, Yasuhide Kawamoto, Fujiko Mitsumoto-Kaseida, Norihito Kaku, Hiroo Hasegawa, Takahiro Takazono, Koichi Izumikawa, Hiroshi Mukae, Katsunori Yanagihara

**Affiliations:** 1 Department of Laboratory Medicine, Nagasaki University Graduate School of Biomedical Sciences, Nagasaki, Japan; 2 Department of Laboratory Medicine, Nagasaki University Hospital, Nagasaki, Japan; 3 Department of Infectious Diseases, Nagasaki University Graduate School of Biomedical Sciences, Nagasaki, Japan; 4 Department of Respiratory Medicine, Nagasaki University Graduate School of Biomedical Sciences, Nagasaki, Japan

**Keywords:** bloodstream infection, antimicrobial resistance, β-lactamase, rapid detection, workflow

## Abstract

Bloodstream infections caused by antimicrobial-resistant Gram-negative bacilli are often difficult to treat. Here, we investigated the performance of a workflow for direct detection of Gram-negative bacilli and extended-spectrum β-lactamase (ESBL) producers in positive blood culture (PBC) specimens using matrix-assisted laser desorption ionization–time-of-flight mass spectrometry (MALDI-TOF MS). Samples were prepared from spiked and clinical PBC specimens using a sample preparation kit, and bacterial identification and cephalosporinase activity assays were performed using MALDI-TOF MS. The results were compared with those obtained using the conventional method, which was performed for colonies as routine microbiological testing. Of the 34 spiked PBC specimens prepared using *Escherichia coli* and *Klebsiella pneumoniae* isolates, all 27 ESBL producers tested positive and seven non-producers tested negative for cephalosporinase activity. Of the 111 clinical PBC specimens analyzed, 99 (89.2%) showed concordance with the conventional method results, with scores ≥ 2.00. Among the 54 Enterobacterales, consisting of *E. coli*, *K. pneumoniae*, *Klebsiella oxytoca*, and *Proteus mirabilis* isolates identified in the directly prepared samples, 14 were ESBL producers in the colonies. Of the 14 ESBL producers, 11 (78.6%) were correctly identified as cephalosporinase producers in the directly prepared samples using MALDI-TOF MS. The time required for direct identification (8.4 h) was significantly shorter than that required for conventional identification (15.0 h). Moreover, the time required for cephalosporinase activity detection in the directly prepared samples (9.2 h) was significantly shorter than that required for phenotypic ESBL detection in colonies (32.3 h). This study demonstrates the performance of the workflow for direct detection of Gram-negative bacilli and cephalosporinase activity in ESBL producers from PBC specimens using MALDI-TOF MS.

## Introduction

Bacteria within Enterobacterales can cause serious bloodstream infections (BSIs) and result in considerable mortality (Kosai et al., 2020, 2023). Third-generation cephalosporin resistance in *Escherichia coli* and extended-spectrum β-lactamase (ESBL)-producing *Klebsiella pneumoniae* are reportedly associated with high mortality rates among patients with bacteremia (Daneman et al., 2023; Li et al., 2023). In our previous studies on Gram-negative bacteremia in Japan, ESBL producers were identified in 21.0–24.3% of *E. coli* isolates and 5.1–6.7% of *Klebsiella* spp. isolates, whereas no carbapenem resistance was observed among them (Kosai et al., 2020, 2023). Although early appropriate antibiotic therapy is essential for patients with BSIs caused by ESBL producers (Tumbarello et al., 2007), a previous study reported that ESBL production is associated with delayed effective therapy and increased mortality rates (Schwaber and Carmeli, 2007). Therefore, the rapid detection of ESBL producers is crucial for prompt and effective therapy for BSIs.

Matrix-assisted laser desorption ionization–time-of-flight mass spectrometry (MALDI-TOF MS) is a proteomic analysis–based method that enables rapid and accurate bacterial identification in clinical settings (Calderaro and Chezzi, 2024). In addition to standard identification using colonies of isolates after subculturing, identification can be made directly from a positive blood culture (PBC) specimen using an MBT Sepsityper Kit, which involves the simple collection of bacterial pellets that can be subjected to MALDI-TOF MS analysis. The use of this kit shortens the processing time by several hours or even days and provides high identification reliability, particularly for Gram-negative bacilli (Morgenthaler and Kostrzewa, 2015).

The MALDI Biotyper Selective Testing of Antibiotic Resistance–β-Lactamase (MBT STAR-BL) assay can detect β-lactamase activity within a few hours by analyzing hydrolysis of β-lactam antibiotics using MALDI-TOF MS (Kawamoto et al., 2018; Ota et al., 2021; Sparbier et al., 2016). We previously reported its performance in detecting cephalosporinase activity among *E. coli* and *K. pneumoniae* colonies (Kawamoto et al., 2019). Furthermore, this method has been applied to detect β-lactamase activity in samples prepared directly from PBC specimens (Lee et al., 2018; Ota et al., 2023).

Here, we aimed to evaluate the performance of a workflow for direct detection of Gram-negative bacilli and cephalosporinase activity within PBC specimens using MALDI-TOF MS. This involved bacterial identification using samples prepared with an MBT Sepsityper Kit, followed by detection of cephalosporinase activity using the MBT STAR-BL assay. Specifically, we focused on the detection performance of Gram-negative bacilli and ESBL producers.

## Materials and methods

### Routine blood culture

Routine blood cultures were performed using a BacT/ALERT VIRTUO automated blood culture system (bioMérieux, Inc., Durham, NC, USA) with BacT/ALERT FA Plus, SN, and PF Plus culture bottles (bioMérieux, Inc., Durham, NC, USA). Gram staining and subculturing were performed when the bottles tested positive. For subculturing, Nissui Separated Plate Sheep Blood Agar/Chocolate Agar EXII (Nissui Pharmaceutical Co., Ltd., Tokyo, Japan) was used for all bottles while Anaero Columbia Agar with Rabbit Blood (Becton Dickinson, Franklin Lakes, NJ, USA) was additionally used for the positive SN bottles. CHROMagar Candida/BTB agar (prepared media) (Kanto Chemical Co., Inc., Tokyo, Japan) was used when yeast or hyphae were observed upon Gram staining. Bacterial isolates were identified using the MALDI Biotyper system (Bruker Daltonics, Billerica, MA, USA). A small amount of a single colony was applied to a spot on a MALDI target plate for each sample and overlaid with 1 μL of α-cyano-4-hydroxycinnamic acid solution. The dried spots were analyzed using a Microflex LT (Bruker Daltonics, Billerica, MA, USA) instrument with flexControl 3.4 (Bruker Daltonics, Billerica, MA, USA). The spectra were analyzed using the MBT Compass software (Bruker Daltonics, Billerica, MA, USA). Antimicrobial susceptibility and ESBL production were determined using the BD Phoenix M50 (Becton Dickinson, Franklin Lakes, NJ, USA) according to the manufacturer’s instructions. The conventional method used herein involved the identification and antimicrobial susceptibility testing of colonies.

### Specimens for direct detection of pathogen and cephalosporinase activity

Spiked blood culture specimens were used to validate the direct cephalosporinase activity assay. Briefly, a BacT/ALERT FA Plus blood culture bottle was spiked with 5 mL of human whole blood (Kohjin Bio Co., Ltd., Saitama, Japan). Bacteria were suspended in sterile saline to a 1 McFarland standard, diluted appropriately, and inoculated into the bottle to achieve a final concentration of approximately 1,000 CFU/mL, after which the bottle was cultured in a BacT/ALERT VIRTUO. Once a positive signal was obtained, the specimens were subjected to subsequent processes. **Table 1** shows the bacteria used for the spiked blood culture specimens. Bacterial profiles were determined in our previous study (Kawamoto et al., 2019).

**Table 1:** Bacterial isolates used for spiked blood culture specimens.

Bacterial species	ESBL genotype^a^	MIC for CTX (μg/mL)^a^	ESBL production^a^	CTX hydrolysis^a^	N
*Escherichia coli*	*bla*_CTX-M-1_ group	> 32	Positive	Positive	2
	*bla*_CTX-M-1_ group, *bla*_TEM_	> 32	Positive	Positive	1
	*bla*_CTX-M-9_ group	> 32	Positive	Positive	6
	*bla*_CTX-M-9_ group, *bla*_TEM_	> 32	Positive	Positive	6
	None	≤ 1	Negative	Negative	3
*Klebsiella pneumoniae*	*bla*_CTX-M-1_ group, *bla*_SHV_	> 32	Positive	Positive	3
	*bla*_CTX-M-1_ group, *bla*_TEM_	> 32	Positive	Positive	1
	*bla*_CTX-M-1_ group, *bla*_SHV_, *bla*_TEM_	> 32	Positive	Positive	2
	*bla*_CTX-M-2_ group, *bla*_SHV_	> 32	Positive	Positive	3
	*bla*_CTX-M-9_ group	> 32	Positive	Positive	1
	*bla*_CTX-M-9_ group, *bla*_TEM_	> 32	Positive	Positive	2
	None	≤ 1	Negative	Negative	4

ESBL, extended-spectrum β-lactamase; MIC, minimum inhibitory concentration; CTX, cefotaxime.

a Bacterial profiles were determined using colonies in our previous study (Kawamoto et al., 2019).

**Figure 1:**
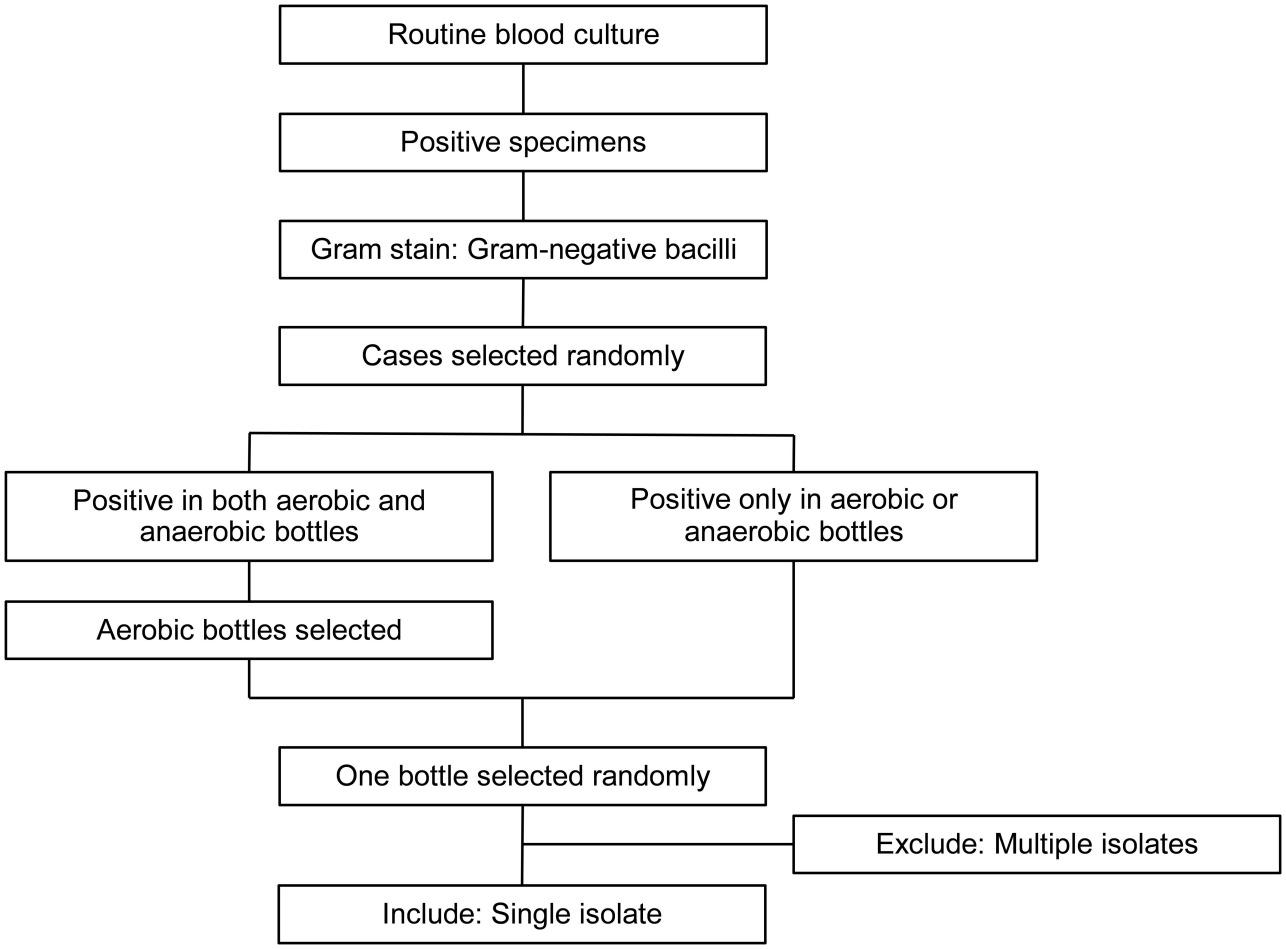
Flow diagram of specimen selection. Positive blood culture specimens were subjected to Gram staining and those with Gram-negative bacilli were randomly selected. For specimens that were positive in both aerobic and anaerobic bottles, one aerobic bottle was randomly selected. For specimens that were positive only in aerobic or anaerobic bottles, one bottle was randomly selected. Only bottles with single isolates were included, whereas those with multiple isolates were excluded.

Clinical PBC specimens were obtained through routine blood cultures. **Figure 1** shows a flow diagram of specimen selection. PBC specimens were randomly selected from those in which Gram-negative bacilli were observed on Gram staining. If Gram-negative bacilli were observed in both aerobic and anaerobic bottles from a single patient, an aerobic bottle was selected. If Gram-negative bacilli were found in multiple bottles of the same type, one bottle was randomly selected. Specimens were collected at Nagasaki University Hospital between June 2022 and January 2024. Specimens in which multiple bacterial genera or species were detected using routine colony testing were excluded from the study.

### Direct detection of pathogens and cephalosporinase activity using MALDI-TOF MS

Spiked and clinical PBC specimens were processed using the MBT Sepsityper Kit (Bruker Daltonics, Billerica, MA, USA). Briefly, 1 mL of each specimen was mixed with 200 μL of lysis buffer and centrifuged for 2 min at 13,000 rpm. After the supernatant was removed, 1 mL of wash buffer was added, and the pellet was resuspended. The suspension was centrifuged for 1 min at 13,000 rpm. The supernatant was removed, and the pellet was subjected to subsequent analysis.

Bacterial identification using a small amount of pellet was performed similarly to routine testing of colonies. The MBT STAR-BL assay using the MBT STAR-Cepha IVD Kit (Bruker Daltonics, Billerica, MA, USA) was performed when *E. coli*, *K. pneumoniae, Klebsiella oxytoca*, and *Proteus mirabilis*, which are species specified for ESBL testing by the Clinical and Laboratory Standards Institute (CLSI) document M100-ED35, were identified in directly prepared samples. Briefly, the residual pellet was suspended and mixed with 50 μL of MBT STAR-Cepha Antibiotic Reagent (Bruker Daltonics, Billerica, MA, USA) and incubated for 35 min at 35 ± 2°C with agitation at 900 rpm. The sample was centrifuged for 2 min at 13,000 rpm, and 1 μL of the supernatant was applied to a spot on a MALDI target plate in duplicate for each sample. The spots were dried and overlaid with 1 μL of the MBT STAR-BL Matrix solution (Bruker Daltonics, Billerica, MA, USA). The dried spots were analyzed using a Microflex LT instrument with flexControl 3.4. The spectra were analyzed using the MBT Compass software with the STAR-BL module (Bruker Daltonics, Billerica, MA, USA). The logRQ value (a measure of hydrolysis efficiency) was calculated automatically. Normalized logRQ values > 0.22 or < 0.08 indicated positive or negative results, respectively, while those between 0.08 and 0.22 were considered indeterminate.

### Detection of carbapenemase genes

For colonies of *E. coli*, *K. pneumoniae*, *klebsiella variicola*, *K. oxytoca*, and *P. mirabilis* isolates from the clinical PBC specimens, carbapenemase genes were examined using Xpert Carba-R (Cepheid, Sunnyvale, CA, USA), which can detect *bla*_KPC_, *bla*_NDM_, *bla*_VIM_, *bla*_OXA-48_, and *bla*_IMP_, according to the manufacturer’s instructions.

### Statistical analysis

The time from PBC to bacterial identification and the time from PBC to the detection of cephalosporinase activity or ESBL production were compared using the Mann–Whitney U test. Data were analyzed using GraphPad Prism v10.6.0 (GraphPad Software, Inc., San Diego, CA, USA), and the results were considered statistically significant at P < 0.05.

## Results

**Figure 2:**
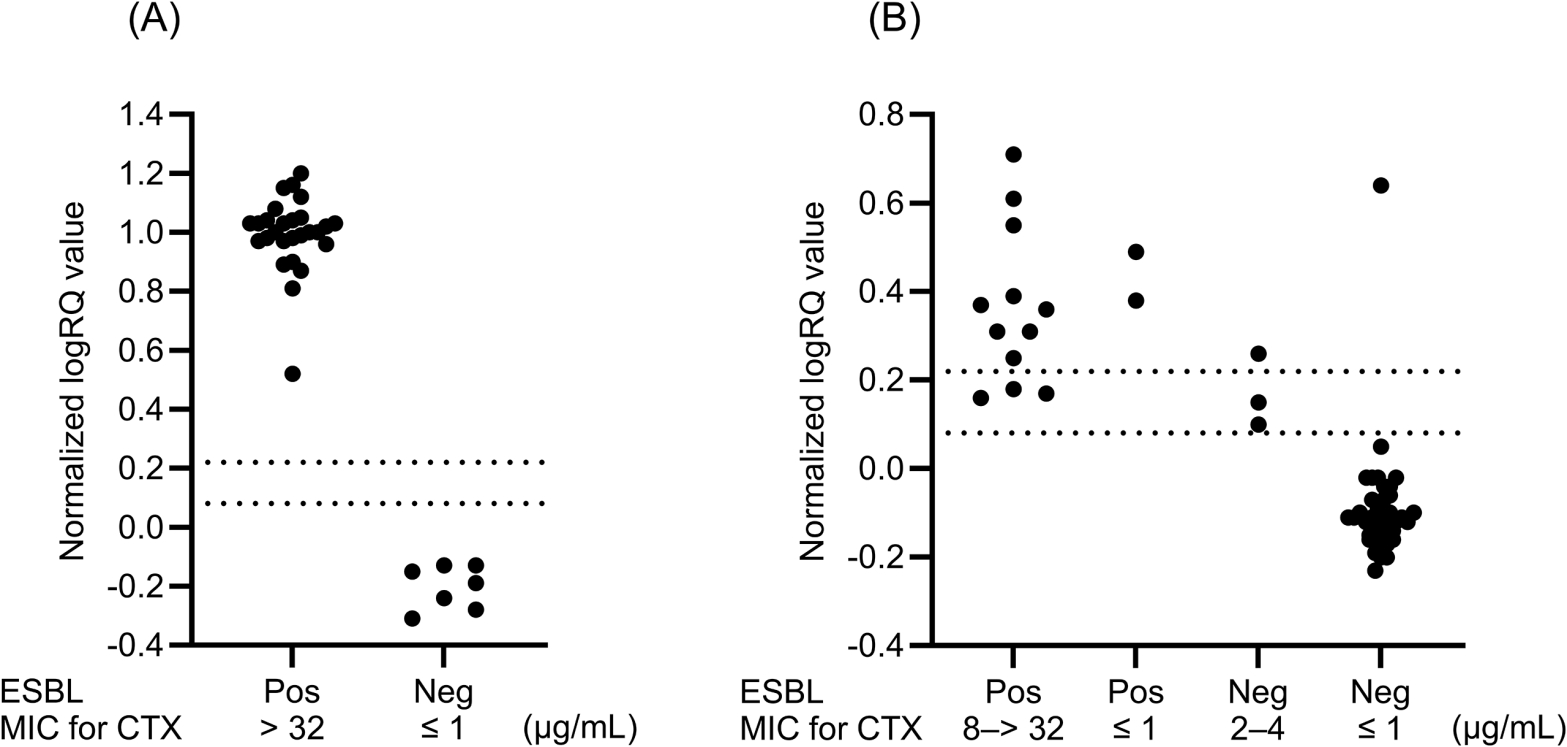
Distributions of normalized logRQ values for cephalosporinase activity in samples obtained from spiked blood culture specimens (n = 34) **(A)** and clinical blood culture specimens (n = 54) **(B)**. The isolates in clinical specimens consist of *E*. *coli*, *K*. *pneumoniae*, *K*. *oxytoca*, and *P. mirabilis*. Normalized logRQ values of > 0.22 and < 0.08 indicate positive and negative results, respectively. Values between 0.08 and 0.22 were considered indeterminate. ESBL, extended-spectrum β-lactamase; MIC, minimum inhibitory concentration; Pos, positive; Neg, negative.

A total of 34 isolates were used to prepare the spiked PBC specimens (**Table 1**). **Figure 2A** shows the distribution of normalized logRQ values for the samples prepared using the MBT Sepsityper Kit. All 27 ESBL producers tested positive for cephalosporinase activity, whereas seven non-producers tested negative.

For clinical specimen analysis, 111 PBC specimens were processed using the MBT Sepsityper Kit. The results of direct bacterial identification of the prepared samples were compared with those obtained from colony identification after subculturing the PBC specimens. **Table 2** presents the concordant identification results with scores ≥ 2.00 between the direct and conventional methods. Of the 111 samples tested, 99 (89.2%) showed concordance with the results obtained using the conventional method. Among the 99 samples, the most prevalent pathogen was *E. coli* (33 isolates, 33.3%), followed by *Enterobacter cloacae* complex (15 isolates, 15.2%), and *K. pneumoniae* (13 isolates, 13.1%). **Table 3** shows samples with identifications that had score < 2.00 or were undetectable by the direct and/or conventional methods. Of these 12 samples, 10 (two *E. coli*, one *K. pneumoniae*, one *P. mirabilis*, four *P. aeruginosa*, one *Stenotrophomonas maltophilia*, and one *Stenotrophomonas* spp.) were identified concordantly between the direct and conventional methods, but the scores were < 2.00 in the direct and/or conventional methods.

**Table 2:** Concordant bacterial identification with score ≥ 2.00 between direct and conventional methods.

Identification	N
*Aeromonas hydrophilia*	1
*Aeromonas* spp.	1
*Bacteroides fragilis*	1
*Citrobacter freundii*	2
*Citrobacter koseri*	1
*Enterobacter cloacae* complex	15
*Escherichia coli*	33
*Escherichia hermannii*	1
*Haemophilus influenzae*	1
*Klebsiella aerogenes*	7
*Klebsiella oxytoca*	6
*Klebsiella pneumoniae*	13
*Morganella morganii*	1
*Proteus mirabilis*	2
*Providencia rettgeri*	1
*Pseudomonas aeruginosa*	7
*Raoultella ornithinolytica*	3
*Serratia marcescens*	3

**Table 3:** Insufficient identification results.

Identification (score)	
Direct method	Conventional method
*Escherichia coli* (1.98)	*Escherichia coli* (2.28)
*Escherichia coli* (1.97)	*Escherichia coli* (2.26)
*Klebsiella pneumoniae* (1.97)	*Klebsiella pneumoniae* (2.17)
*Klebsiella pneumoniae* (1.96)	*Klebsiella variicola* (2.05)
*Proteus mirabilis* (1.94)	*Proteus mirabilis* (2.51)
*Pseudomonas aeruginosa* (1.96)	*Pseudomonas aeruginosa* (2.11)
*Pseudomonas aeruginosa* (1.61)	*Pseudomonas aeruginosa* (2.14)
*Pseudomonas aeruginosa* (1.50)	*Pseudomonas aeruginosa* (1.96)
*Pseudomonas aeruginosa* (1.48)	*Pseudomonas aeruginosa* (2.54)
*Stenotrophomonas maltophilia* (1.85)	*Stenotrophomonas maltophilia* (2.30)
*Stenotrophomonas* spp. (1.62)	*Stenotrophomonas* spp. (2.15)
No peaks found	*Citrobacter koseri* (2.42)

Among the Enterobacterales isolates with scores ≥ 2.00 in samples prepared directly from PBC specimens, we focused on 54 isolates, consisting of 33 *E. coli*, 13 K*. pneumoniae*, six *K. oxytoca*, and two *P. mirabilis* isolates, which were specified for ESBL testing by the CLSI document M100-ED35, in the cephalosporinase activity assay. Of these, 14 (12 *E. coli* and 2 K*. oxytoca*) were finally identified using colonies as phenotypic ESBL producers. Of these, 11 (nine *E. coli* and two *K. oxytoca*) (78.6%) were correctly identified as cephalosporinase producers using the MBT STAR-BL assay for the directly prepared samples (**Figure 2B**). Four isolates (two *E. coli*, one *K. pneumoniae*, and one *P. mirabilis*) with scores ranging from 1.94 to 1.98 for direct detection but ≥ 2.00 for colonies (**Table 3**) included one ESBL and three non-ESBL producers, which correctly tested positive and negative, respectively, in the cephalosporinase activity assay using the direct MBT STAR-BL assay. We observed three indeterminate results for ESBL producers and two false positives and two indeterminate results for non-ESBL producers in the MBT STAR-BL assay using the directly prepared samples (**Figure 2B**).

All 59 Enterobacterales, consisting of *E. coli*, *K. pneumoniae*, *K.* variicola, *K. oxytoca*, and *P. mirabilis* isolates, examined in this study showed minimum inhibitory concentrations of ≤ 1 µg/mL for meropenem and tested negative for carbapenemase genes on the Xpert Carba-R.

**Figure 3:**
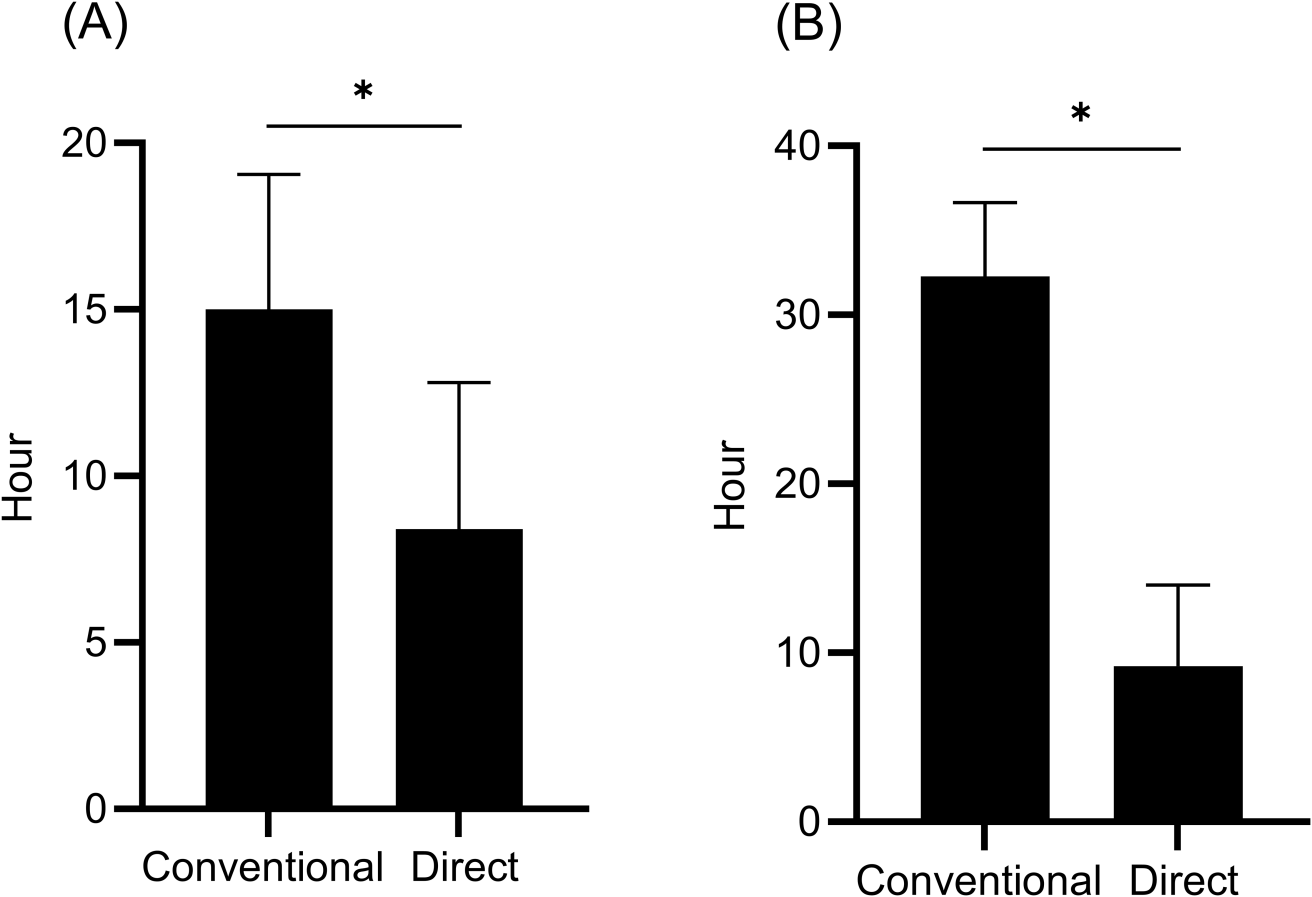
Time from positive blood culture to bacterial identification (n = 97) **(A)** and time from positive blood culture to detection of ESBL production (conventional) or cephalosporinase activity (direct) (n = 53) **(B)**. The conventional method was performed as a routine test using colonies of isolates from positive blood culture specimens, whereas the direct method was performed using samples prepared from positive blood culture specimens using a sample preparation kit. Data are expressed as median with interquartile range. *P < 0.0001.

The times required for direct identification and cephalosporinase activity detection were not available for samples containing one *E. coli* and one *Klebsiella aerogenes* isolate. Consequently, 97 samples for direct identification and 53 samples for cephalosporinase activity were included in the analysis of the differences in the required time. The time required for direct identification (8.4 h) was significantly shorter than that for conventional identification (15.0 h) (**Figure 3A**). In addition, significantly less time was required to detect cephalosporinase activity within the directly prepared samples (9.2 h) than to detect ESBL production using phenotypic tests for colonies (32.3 h) (**Figure 3B**).

## Discussion

This study demonstrated the performance of a workflow using MALDI-TOF MS for direct detection of Gram-negative bacilli and subsequent analysis of cephalosporinase activity in Enterobacterales, consisting of *E. coli*, *K. pneumoniae*, *K. oxytoca*, and *P. mirabilis* isolates. Analysis of spiked PBC specimens showed excellent performance and completely distinguished ESBL producers from non-producers based on cephalosporinase activity (**Figure 2A**). All PBC samples containing ESBL producers correctly tested positive for cephalosporinase activity, which is consistent with our previous analysis for colonies of identical isolates (Kawamoto et al., 2019). In earlier Japanese surveillance, including data from our hospital, *bla*_CTX-M_ group 9 (77.3%) was predominant among *E. coli*, followed by group 1 (18.2%), whereas in *Klebsiella* spp., group 1 (50.0%) was more common than group 9 (37.5%) (Kosai et al., 2023). The isolates used for the spiked blood culture specimens in this study represented the major *bla*_CTX_-M subgroups observed in our clinical setting.

In the analysis of clinical PBC specimens obtained from routine blood culture testing, 89.2% (99/111) showed concordance between the direct and conventional methods for identification, with scores ≥ 2.00 (**Table 2**), a finding that is similar to the results of a previous report (89.6% in Gram-negative bacteria) (Morgenthaler and Kostrzewa, 2015). Of the remaining 11 samples that showed scores ≥ 2.00 using the conventional methods, six showed concordant results with scores ≥ 1.80 using the direct identification (**Table 3**), indicating that 94.6% (105/111) of the samples exhibited concordance at the genus level. The low scores observed in the directly prepared samples may be attributable to nonspecific peaks associated with blood culture, such as those derived from leukocyte proteins (Morgenthaler and Kostrzewa, 2015).

Among 54 Enterobacterales isolates with scores ≥ 2.00 from positive blood culture specimens, consisting of *E. coli*, *K. pneumoniae*, *K. oxytoca*, and *P. mirabilis* isolates, 14 (25.9%) were identified as phenotypic ESBL producers using colonies. In the MBT STAR-BL assay, 78.6% (11/14) of the ESBL producers were detected as cephalosporinase producers. This result is consistent with a previous study showing that 71.4% of ESBL producers demonstrated cefotaxime hydrolysis in a direct MBT STAR-BL assay for samples prepared using a saponin-based extraction method from blood culture broths (Lee et al., 2018). In another study, the MBT STAR-Cepha kit showed the performance with an AUC of 0.81 for detecting ESBL producers, and CTX-resistant strains mainly harboring CTX-M-type ESBLs were accurately identified (Ota et al., 2023).

In our study, three of the 14 ESBL producers showed indeterminate results in the MBT STAR-BL assay (**Figure 2B**). Therefore, isolates with indeterminate results should be regarded as ESBL producers until the final results for the colonies after subculturing are obtained. In contrast, all isolates with negative MBT STAR-BL assay results were non-ESBL producers (**Figure 2B**). This indicates that negative cephalosporinase activity results may exclude ESBL production. We could not identify the cause of the two false positives and two indeterminate results in the cephalosporinase activity assay for non-ESBL producers. Because colonies obtained from the same clinical PBC specimens tested negative for carbapenemase genes, other cephalosporinases such as AmpC might be a cause. Mutations in the chromosomal *ampC* promoter and attenuator regions can cause *ampC* overexpression in *E. coli*, whereas plasmid-mediated *ampC* may contribute to AmpC β-lactamase activity in *E. coli* and *Klebsiella* spp (Jacoby and Munoz-Price, 2005; Peter-Getzlaff et al., 2011).

Furthermore, the workflow evaluated in this study significantly reduced the median time required for bacterial identification (from 15.0 to 8.4 h) and detection of ESBL producers, which was assessed based on cephalosporinase activity (from 32.3 to 9.2 h). A recent study revealed that inappropriate antimicrobial treatment at 12 hours after blood culture collection was associated with increased 30-day mortality in patients with BSIs (Van Heuverswyn et al., 2023). These results indicate that our accelerated workflow may allow for earlier administration of appropriate antimicrobial therapy, potentially leading to improved clinical outcomes. Further studies are needed to examine the clinical impact of this rapid detection workflow for pathogens and cephalosporinase activity using MALDI-TOF MS.

There are some limitations in this study. First, this study included relatively small sample sizes for the analyses of spiked (n = 34) and clinical (n = 111) specimens. Second, STAR-BL assay was performed for selected Enterobacterales, consisting of *E. coli*, *K. pneumoniae*, *K. oxytoca*, and *P. mirabilis*. Third, resistance mechanisms other than ESBLs and carbapenemases, such as AmpC, were not evaluated in the analysis of cephalosporinase activity. Furthermore, we observed indeterminate results in addition to false positives. These factors should be considered when interpreting findings and applying this method clinically.

In conclusion, this study demonstrated the performance of a workflow using an MALDI-TOF MS system to detect Gram-negative bacilli and cephalosporinase activity in ESBL-producing Enterobacterales, consisting of *E. coli*, *K. pneumoniae*, *K. oxytoca*, and *P. mirabilis*. Further studies are required to assess how this workflow can support the early and appropriate antimicrobial selection in patients with BSIs.

## Data Availability

The raw data supporting the conclusions of this article will be made available by the authors, without undue reservation.

## References

[B1] Calderaro A. Chezzi C. (2024). MALDI-TOF MS: a reliable tool in the real life of the clinical microbiology laboratory. Microorganisms. 12, 322. doi: 10.3390/microorganisms12020322, PMID: 38399726 PMC10892259

[B2] Daneman N. Fridman D. Johnstone J. Langford B. J. Lee S. M. MacFadden D. M. . (2023). Antimicrobial resistance and mortality following *E. coli* bacteremia. EClinicalMedicine. 56, 101781. doi: 10.1016/j.eclinm.2022.101781, PMID: 36618891 PMC9813674

[B3] Jacoby G. A. Munoz-Price L. S. (2005). The new β-lactamases. N. Engl. J. Med. 352, 380–391. doi: 10.1056/NEJMra041359, PMID: 15673804

[B4] Kawamoto Y. Kosai K. Yamakawa H. Kaku N. Uno N. Morinaga Y. . (2018). Performance evaluation of the MALDI Biotyper Selective Testing of Antibiotic Resistance-β-Lactamase (MBT STAR-BL) assay for the detection of IMP metallo-β-lactamase activity in *Enterobacteriaceae*. Diagn. Microbiol. Infect. Dis. 92, 275–278. doi: 10.1016/j.diagmicrobio.2018.06.018, PMID: 30041842

[B5] Kawamoto Y. Kosai K. Yamakawa H. Kaku N. Uno N. Morinaga Y. . (2019). Detection of extended-spectrum β-lactamase (ESBL)-producing *Enterobacteriaceae* using the MALDI Biotyper Selective Testing of Antibiotic Resistance-β-Lactamase (MBT STAR-BL) assay. J. Microbiol. Methods 160, 154–156. doi: 10.1016/j.mimet.2019.03.019, PMID: 30904555

[B6] Kosai K. Yamagishi Y. Hashinaga K. Nakajima K. Mikamo H. Hiramatsu K. . (2020). Multicenter surveillance of the epidemiology of gram-negative bacteremia in Japan. J. Infect. Chemother. 26, 193–198. doi: 10.1016/j.jiac.2019.11.003, PMID: 31932213

[B7] Kosai K. Yamagishi Y. Mikamo H. Ishii Y. Tateda K. Yanagihara K. (2023). Epidemiological analysis and antimicrobial susceptibility profile of Gram-negative bacilli that cause bacteremia in Japan. J. Infect. Chemother. 29, 1091–1096. doi: 10.1016/j.jiac.2023.08.012, PMID: 37597749

[B8] Lee A. W. T. Lam J. K. S. Lam R. K. W. Ng W. H. Lee E. N. L. Lee V. T. Y. . (2018). Comprehensive evaluation of the MBT STAR-BL module for simultaneous bacterial identification and β-lactamase-mediated resistance detection in Gram-negative rods from cultured isolates and positive blood cultures. Front. Microbiol. 9. doi: 10.3389/fmicb.2018.00334, PMID: 29527202 PMC5829630

[B9] Li D. Huang X. Rao H. Yu H. Long S. Li Y. . (2023). *Klebsiella pneumoniae* bacteremia mortality: a systematic review and meta-analysis. Front. Cell. Infect. Microbiol. 13. doi: 10.3389/fcimb.2023.1157010, PMID: 37153146 PMC10159367

[B10] Morgenthaler N. G. Kostrzewa M. (2015). Rapid identification of pathogens in positive blood culture of patients with sepsis: review and meta-analysis of the performance of the sepsityper kit. Int. J. Microbiol. 2015, 827416. doi: 10.1155/2015/827416, PMID: 26000017 PMC4426779

[B11] Ota Y. Furuhashi K. Hirai N. Ishikawa J. Nagura O. Yamanaka K. . (2021). Evaluation of MBT STAR-Cepha and MBT STAR-Carba kits for the detection of extended-spectrum β-lactamases and carbapenemase producing microorganisms using matrix-assisted laser desorption/ionization time-of-flight mass spectrometry. J. Microbiol. Methods 183, 106166. doi: 10.1016/j.mimet.2021.106166, PMID: 33600876

[B12] Ota Y. Furuhashi K. Hirai N. Nagao Y. Ishikawa J. Nagura O. . (2023). Utility of the MBT STAR-Cepha kit in detecting extended-spectrum β-lactamase producers in clinical urine samples and positive blood cultures using matrix-assisted laser desorption/ionization time-of-flight mass spectrometry. J. Microbiol. Methods 211, 106756. doi: 10.1016/j.mimet.2023.106756, PMID: 37285970

[B13] Peter-Getzlaff S. Polsfuss S. Poledica M. Hombach M. Giger J. Bottger E. C. . (2011). Detection of AmpC β-lactamase in Escherichia coli: Comparison of three phenotypic confirmation assays and genetic analysis. J. Clin. Microbiol. 49, 2924–2932. doi: 10.1128/JCM.00091-11, PMID: 21653764 PMC3147754

[B14] Schwaber M. J. Carmeli Y. (2007). Mortality and delay in effective therapy associated with extended-spectrum β-lactamase production in Enterobacteriaceae bacteraemia: a systematic review and meta-analysis. J. Antimicrob. Chemother. 60, 913–920. doi: 10.1093/jac/dkm318, PMID: 17848376

[B15] Sparbier K. Schubert S. Kostrzewa M. (2016). MBT-ASTRA: A suitable tool for fast antibiotic susceptibility testing? Methods. 104, 48–54. doi: 10.1016/j.ymeth.2016.01.008, PMID: 26804565

[B16] Tumbarello M. Sanguinetti M. Montuori E. Trecarichi E. M. Posteraro B. Fiori B. . (2007). Predictors of mortality in patients with bloodstream infections caused by extended-spectrum-β-lactamase-producing Enterobacteriaceae: importance of inadequate initial antimicrobial treatment. Antimicrob. Agents. Chemother. 51, 1987–1994. doi: 10.1128/AAC.01509-06, PMID: 17387156 PMC1891412

[B17] Van Heuverswyn J. Valik J. K. Desiree van der Werff S. Hedberg P. Giske C. Naucler P. (2023). Association between time to appropriate antimicrobial treatment and 30-day mortality in patients with bloodstream infections: a retrospective cohort study. Clin. Infect. Dis. 76, 469–478. doi: 10.1093/cid/ciac727, PMID: 36065752 PMC9907509

